# Supplementary material for: Exploring the Diversity of Microbial Communities Associated with Two Anopheles Species During Dry Season in an Indigenous Community from the Colombian Amazon
Source: Insects. 2025 Mar 4;16(3):269. doi: 10.3390/insects16030269 (PMC11942818; doi:10.3390/insects16030269)
Supplement: Supplementary file 1 [file insects-16-00269-s001.zip › insects-3354862-supplementary.pdf]

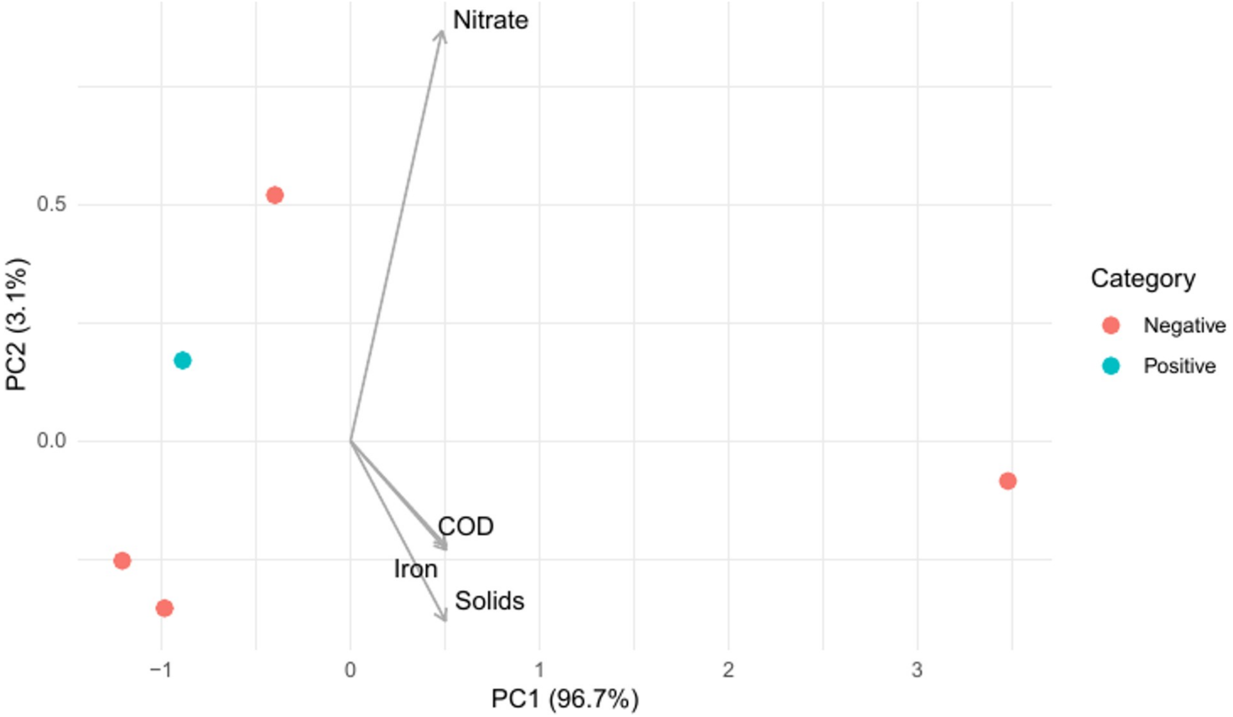

Figure S1. PCA of the five water bodies sampled for COD, Nitrate, Iron, and fixed and volatile solids in S.P.L.-Leticia, Amazonas. Copper was excluded from the analysis as all values were under 0.092 mg/L (detection limit). Sites without presence of anopheline larvae or pupae are represented in pink, while the fishing pond, positive for anopheline larvae and pupae is represented in blue.

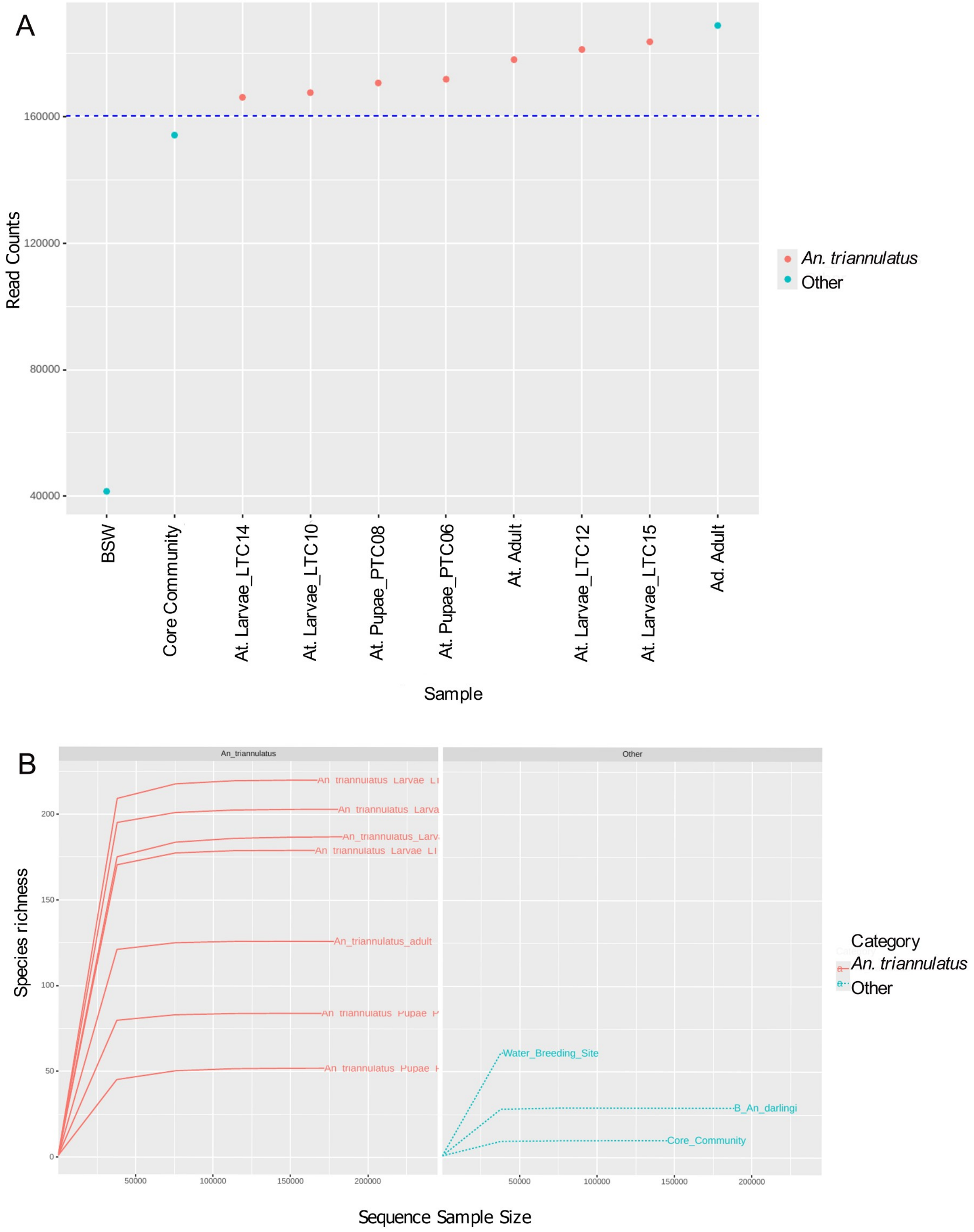

Figure S2. Library size (A) and rarefaction curves (B) of anophelines and breeding site collected in San Pedro de los Lagos, Amazonas. The Core Community was used as a control (Microbial Community Standard from ZYMO).

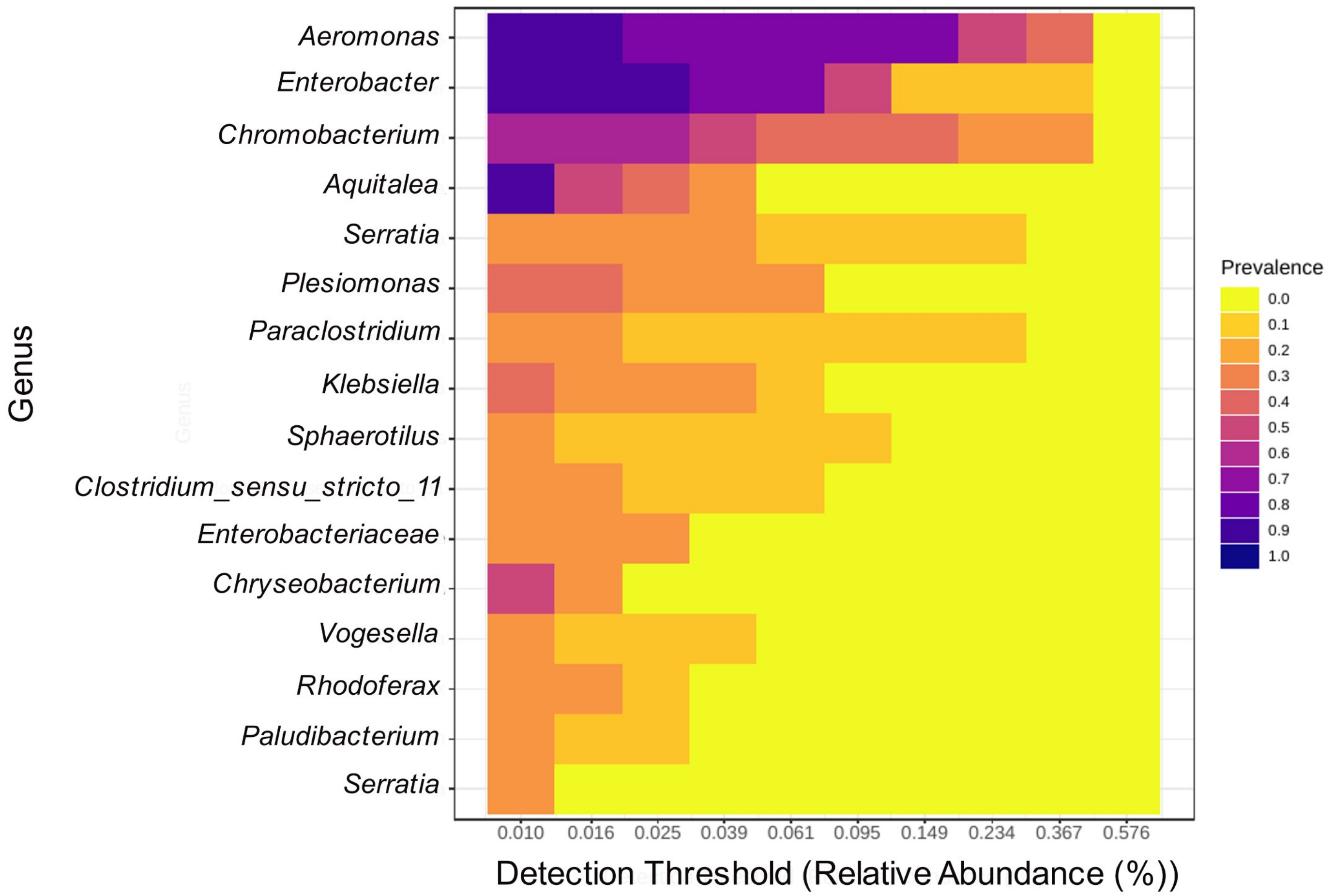

Figure S3. Core microbiome at the genus level recovered from the total DNA of anopheline specimens. Higher prevalence of the bacteria is associated with purple color, while low prevalence is presented in yellow.

Table S1. Molecular markers and primers used for detection of five endosymbionts and DNA barcoding for the identification of *Anopheles* specimens in the Colombian Amazon.

Species	Genes	Primers (5′–3′)		Fragment size (bp)	Reference
Wolbachia	*w*sp	*w*sp81F	TGGTCCAATAAGTGATGAAGAAAC	610	Duron et al. (2008)
		*w*sp691R	AAAAATTAAACGCTACTCCA		
Cardinium	16S rDNA	CLO-f1	GGAACCTTACCTGGGCTAGAATGTATT	466	
		CLO-r1	GCCACTGTCTTCAAGCTCTACCAAC		
Spiroplasma sp.	16S rDNA	SPIRO1	GGGTGAGTAACACGTATCT	1024	
		SPIRO2	CCTTCCTCTAGCTTACACTA		
Arsenophonus sp.	16S rDNA	ArsF	GGGTTGTAAAGTACTTTCAGTCGT	804	
		ArsR2	GTAGCCCTRCTCGTAAGGGCC		
	23S rRNA	Ars23S-1	CGTTTGATGAATTCATAGTCAAA	790	Gosh et al. (2015)
		Ars23S-2	GGTCCTCCAGTTAGTGTTACCCAAC		
Microsporidia	ss18S	ss18SF	GTTGATTCTGCCTGACGT	1474	Vivero-Gomez et al. (2021)
		ss1492R	GGTTACCTTGTTACGACTT		
Anopheles mosquitoes	Cytochrome oxidase subunit I	LCO1490	GGTCAACAAATCATAAAGATATTGG	710	Folmer et al. (1994)
		HCO2198	TAAACTTCAGGGTGACCAAAAAATCA		

Table S2. Identities by classical taxonomy and molecular identification of the *Anopheles* mosquitoes collected in different environments, type of analysis conducted, and number of individuals processed per life stage.

Identity by Classical Taxonomy	Identity by DNA Barcoding	Life stage	Number of individuals
Anopheles sp.	*An. darlingi*	Larva	1[a, e, g]
An. darlingi		Adult	6[b, e, f, g]
			10[b, f, g]
An. triannulatus	*An. triannulatus*	Larva	5[c, e, g]
			31[c, g]
			20[c, f, g]
		Pupa	5[c, e, f, g]
			5[c, f, g]
			10[c, g]
Anopheles sp.		Adult	11[d, e, g]
An. triannulatus			3[c, f, g]
Anopheles sp.			9[d, g]
An. nuneztovari	*An. dunhami*	Adult	1[d, e, g]
An. darlingi	*An. squamifemur*	Adult	1[d, e, g]
Anopheles sp.	*An. squamifemur*	Adult	1[d, e, g]
Anopheles sp.	*Anopheles* sp.	Adult	6[d, g]
**Total**			125

[a] Flooded canoe at fishing pond
[b] Intra- and peridomestic areas
[c] Fishing pond
[d] Amazonian secondary forest
[e] Species confirmation using the COX1 marker
[f] Next Generation Sequencing
[g] Endosymbiont-specific PCR
